# Isolation and Identification of Antioxidant Peptides Derived from Cricket (*Gryllus bimaculatus*) Protein Fractions

**DOI:** 10.3390/insects14080674

**Published:** 2023-07-29

**Authors:** Olumide Oluwatoyosi Fashakin, Pipat Tangjaidee, Kridsada Unban, Wannaporn Klangpetch, Tabkrich Khumsap, Korawan Sringarm, Saroat Rawdkuen, Suphat Phongthai

**Affiliations:** 1 Master’s Degree Program in Food Science and Technology (International Program), Faculty of Agro-Industry, Chiang Mai University, Chiang Mai 50100, Thailand; olumidejamesfashakin@gmail.com; 2 Division of Food Science and Technology, Faculty of Agro-Industry, Chiang Mai University, Chiang Mai 50100, Thailand; pipat.t@cmu.ac.th (P.T.); kridsada.u@cmu.ac.th (K.U.); wannaporn.u@cmu.ac.th (W.K.); tabkrich.khumsap@cmu.ac.th (T.K.); 3 The Cluster of Agro Bio-Circular-Green Industry (Agro BCG), Chiang Mai University, Chiang Mai 50100, Thailand; korawan.s@cmu.ac.th; 4 Unit of Innovative Food Packaging and Biomaterials, School of Agro-Industry, Mae Fah Luang University, Chiang Rai 57100, Thailand; saroat@mfu.ac.th

**Keywords:** cricket protein fraction (CPF), antioxidant peptides, fractionation, protein hydrolysate, bioactive peptide

## Abstract

**Simple Summary:**

The consumption of crickets, like that of other insects, has beneficial effects such as improving gut health, ameliorating chronic diseases because of their bioactivity, and aiding food products when crickets are used as a protein ingredient, such as in baked foods. However, it has not been generally accepted globally as an alternative protein to the mainstream. In this study, a new way to utilize cricket protein as a functional ingredient was explored. The antioxidant activity of cricket protein hydrolysates prepared by different proteases was analyzed and the identification of amino acids sequence responsible for the increased bioactivity of peptides after purification was conducted. The results showed that cricket proteins can be an alternate source of edible protein and bioactive peptides in addition to the mainstream.

**Abstract:**

Crickets contain high protein content that can be used to improve nutrition but are less exploited. This study was conducted to isolate different Cricket Protein Fractions including albumin, globulin, glutelin, and prolamin. All fractions were characterized and hydrolyzed by commercial enzymes. The results showed that the glutelin fractions had the highest extraction yields with 53.9 ± 2.12% (*p* < 0.05). Moreover, glutelin hydrolysate fraction prepared by Alcalase with a 16.35 ±0.29% hydrolysis degree was selected for further purification because of their high antioxidant activities, including ABTS radical-scavenging activity (0.44–0.55 µmol Trolox eq./g) and metal chelating activity (1721.99–1751.71 µmol EDTA eq./g). Two active fractions, GA-1 (<3 kDa) and GA-2 (<3 kDa), were collected from the consecutive purification of glutelin hydrolysates, which included processes such as membrane ultrafiltration and gel filtration. The fractions were analyzed by LC-MS/MS to obtain 10 peptides with 3–13 amino acids identified as TEAPLNPK, EVGA, KLL, TGNLPGAAHPLLL, AHLLT, LSPLYE, AGVL, VAAV, VAGL, and QLL with a molecular weight range of 359.23–721.37 Da in the two fractions. The amino acid sequence shows a prevalence of hydrophobic amino acids (50–100%) such as valine and leucine in the peptide chains, accounting for its high antioxidant activity. In conclusion, cricket glutelin hydrolysate prepared by Alcalase can serve as an alternative source of potent edible bioactive peptides in functional food products.

## 1. Introduction

Bioactive peptides are inactive precursors that are hidden components of protein found in different food sources. They can be activated through processes such as enzymatic hydrolysis, gastrointestinal digestion, or fermentation to release the active components [1]. The properties of bioactive peptides, such as their anticancer, antidiabetic, and antioxidant activities, depend on the structure and the sequence of amino acids present in the peptides [2]. Aside from the health benefit of food protein as a result of its active component, it is also critical to mention its influence on food security. Food protein will have an effect on population growth in the next decades because of the rapid increase in the world’s population [3]. Protein demand has increased over the years because of its benefits such as its impact on satiety, weight management, and the stimulation of hunger in both old and young people. These factors will increase the demand for protein in the near future, specifically by almost one-third by 2050 [4].

Cricket protein is an alternative source of protein, like other insect proteins that are less exploited [5]. Crickets are one of the types of popular insects that have high-ranked protein content. They are found in every part of the world and the highest number of species of cricket are usually found in the tropics with warm temperatures, which encourage their development in contrast to other environments [6]. It has been reported that the consumption of crickets is at the high end in countries with the problem of food insecurity. However, the cricket is one of the most consumed insects globally [7]. Crickets are commonly eaten in Thailand, East Africa, and other insect-eating places. Further, regarding the practice of insect consumption in recent times, crickets are the most consumed insects, wherein both adult and young crickets are consumed [6,7]. They contain nutrients such as proteins (approximately 45% of their dry mass) and fats (approximately 26% of their dry mass). Other nutritional contents are amino acids, carbohydrates, vitamins, minerals, etc. [8,9]. Cricket powders serve as additives to increase the protein content of food products and are used as food ingredients [10]. It has been reported that the incorporation of protein hydrolysate from crickets in tortillas increases their sensory characteristics [11]. Recently, the therapeutic potential of crickets (like other insects) has gained increased interest. They have been reported for their antioxidant activity via an up-regulation in antioxidant enzymes such as catalase and glutathione peroxidase [12].

Antioxidants can reduce oxidative stress by inhibiting free radicals, thereby ameliorating chronic diseases associated with aging like cancer and arteriosclerosis [13,14]. As a result, research studies on natural antioxidants have become numerous. The antioxidant peptide is one such natural peptide that has gained popularity. Antioxidants and amino acids are interrelated; free amino acids (such as histidine, arginine, and lysine) present in peptides can inhibit free radicals that cause oxidation [15]. Research has also shown that edible insects generally have high antiradical activity and can also chelate iron ions [16]. The extraction of protein by using the Osborne method is typical for storage proteins, and this method is quite new for the classification of protein in insects, majorly because of limited storage domains. During the extraction of protein, Osborne fractionation is usually employed to allow the extraction of water-soluble albumins, saline-soluble globulin, alkali-soluble glutelin, and alcohol-soluble prolamin, each depending on the solvent used; the solvents are deionized water, NaCl, NaOH, and ethanol, respectively [17,18]. This method allows the extraction of a complex mixture of proteins of different structures and peculiar functional properties [19]. Bioactive peptides often exhibit different antioxidant activities as a result of the amino acid profile in a protein fraction. This is because the amino acid components contribute to its solubility (Osborne) and antioxidant activity [20]. The solubility properties of protein differ among different types of solvents as a result of the pH and ionic strength of each solvent [18]. Enzymatic hydrolysis is a critical step for peptide liberation from protein sources. Evidence has shown that the most therapeutic effect of edible insects is in the form of protein hydrolysates, which contain peptides that inhibit inflammations and ameliorate chronic diseases such as hypertension and diabetes [21]. More precisely, antioxidant peptides are inactive in their native source [14] and have to be released by commercial enzymes such as Alcalase, Flavourzyme, Protamex, Neutrase, etc., as well as by digestive enzymes like trypsin and pepsin [22,23]. According to limited information on the antioxidant peptides derived from the different fractions of cricket protein, this study aimed to determine the extraction yield of cricket protein through Osborne fractionation and the degree of hydrolysis of cricket protein (hydrolyzed with Alcalase, Flavourzyme, and Protamex), and to investigate the antioxidant activity of cricket protein hydrolysate purified by using membrane ultrafiltration and chromatography techniques. Finally, the amino acid sequence of the fractions obtained after gel filtration was identified.

## 2. Materials and Methods

### 2.1. Materials

Fresh crickets (*Gryllus bimaculatus*) were purchased from a local cricket farm in Chaing Mai, Thailand. Chemical reagents used for Osborne fractionation such as sodium chloride (NaCl), sodium hydroxide (NaOH), hydrochloric acid (HCl), 2,2′-azino-bis (3-ethylbenzothiazoline-6-sulfonic acid; ABTS), ferrozine, Trolox, ethylenediaminetetraacetic acid (EDTA), ferrous sulfate (FeSO_4_·7H_2_O)], and Alcalase from *Bacillus licheniformis* (2.4 U/g, EC 3.4.21.14) were all purchased from Sigma Aldrich Chemical Company. Protamex (1.5 AU-A/g) and Flavourzyme (500–1000 LAPU/g) from Novozymes were supported by Brenntag Ingredients (Bangkok, Thailand) Public Company Limited.

### 2.2. Cricket Preparation

Fresh crickets were grinded using a blender and the proximate analysis of the blended sample was conducted following the standard methods as reported in AOAC [24]. The cricket sample (56.15 ± 0.24% crude protein, 4.01 ± 0.59% Ash content, 15.04 ± 0.14% lipid, and 1.25 ± 0.73% moisture content) was kept in a freezer before being used in the protein extraction step.

### 2.3. Cricket Protein Fractions (CPF)

Cricket Protein Fractions (CPF) of albumin, globulin, glutelin, and prolamin were obtained through Osborne fractionation based on their solubility as described by Sardari et al. [17], with a little adjustment. Briefly, 200 g of fresh cricket was mixed with DI water (1:5 *w*/*v*) and extracted sequentially with four solutions: DI water, 5% NaCl, 0.1 M NaOH, and 70% ethanol.

Albumin was extracted by initial mixing with DI water (1:5 *w*/*v*), followed by continuous stirring for 1 h using a magnetic stirrer, and centrifuged at a rate of 5000× *g* for 10 min. Following the same protocol, globulin was extracted with 5% NaCl (500 mL) from the residue obtained after the extraction of albumin, stirred continuously for 1 h, and separated in a centrifuge at 5000× *g* for 10 min. The supernatants of both fractions were obtained, their pH values were adjusted to 4.2, centrifuged to collect the precipitates and they were lyophilized.

**Figure 1:**
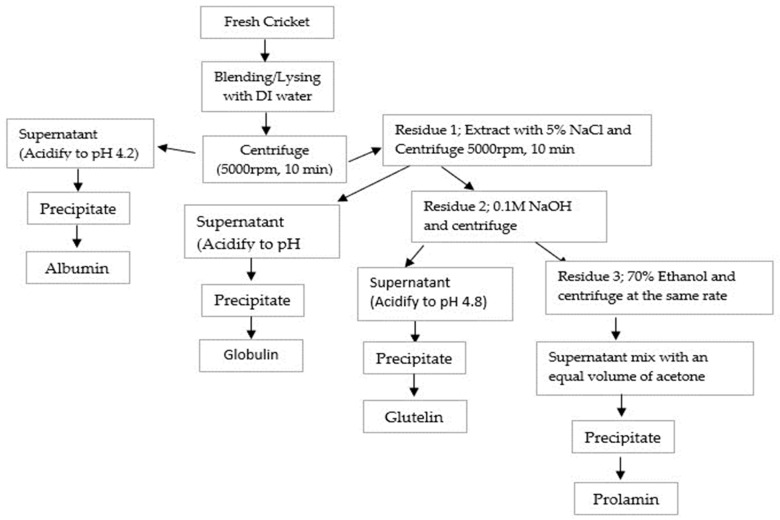
Flowchart of Osborne fractionation of CPF based on solubility.

The residue remaining was allowed to undergo another step of extraction for glutelin with 0.1 M NaOH (500 mL), stirred for 1 h, and centrifuged at 5000× *g* for 10 min. The supernatant was obtained, acidified to pH 4.8 using HCl, centrifuged the second time for 10 min at 5000× *g*, and lyophilized using a freeze dryer. The residue was finally extracted for prolamin using 70% ethanol (500 mL), stirred continuously for 1 h, and centrifuged at 5000× *g* for 10 min. The prolamin supernatant obtained was mixed with acetone twice its volume and allowed to precipitate overnight at −20 °C. Before being lyophilized, the precipitate prolamin was washed with 100 mL DI water, centrifuged, and adjusted to pH 7.0. The powder was collected into a zip-lock bag. After freeze-drying, the yield of each fraction was determined. The extraction processes follow as indicated in Figure 1.

The protein yield (%) and protein recovery (%) were calculated using the formulae below:

i. Protein Yield (PY) in % = (Weight of Extracted Cricket Fraction/Weight of Cricket) × 100
ii. Protein recovery (%) = [(Weight of Extracted Protein x %Protein in Extracted Protein) / (Weight of Cricket Powder Used x %Protein in Cricket Powder)] × 100

### 2.4. Amino Acid Profile of CPF

The amino acid profiles of CPF were determined following the method of the AOAC Official Method of Analysis [24] by using GC-MS.

### 2.5. Protein Pattern by Electrophoresis

The protein patterns of Cricket Protein Fractions were investigated based on the method described by Laemmli et al. [25] using sodium dodecyl sulfate-polyacrylamide gel electrophoresis (SDS-PAGE). Briefly, 15 mg of each fraction was dissolved in 15 mg of the prepared buffer (0.125 M Tris–HCl, pH 6.8, 4% SDS, and 20% glycerol) and heated at a temperature of 100 °C for about 10 min. After cooling, the mixture was loaded onto 12% separation gel. Protein bands were stained with a solution of Coomassie Brilliant Blue R-250 containing 50% methanol and 7.5% acetic acid. The gels were de-stained with 50% methanol and 7.5% acetic acid for 40 min. They were then further de-stained with 5% (*v*/*v*) methanol and 7.5% (*v*/*v*) acetic acid for 20 min and finally washed and dried.

### 2.6. Enzymatic Hydrolysis of Cricket Protein Fractions (CPF)

CPF was hydrolyzed with the protease enzymes Alcalase, Flavourzyme, and Protamex. This was carried out following the methodology described by Aluko [18] with some modifications. Lyophilized protein fractions were dispersed in DI water (1:10 *w*/*v*), homogenized at 25 °C for 30 min, pH-adjusted (to 8.5 at 55 °C for Alcalase and 7.0 at 50 °C for Flavourzyme and Protamex) using 0.5 M NaOH, and stirred continuously while incubating at 55 °C. Alcalase, Flavourzyme, and Protamex were added at the optimal conditions—1:100, 10:100, and 10:100 *w*/*w* (enzyme/sample), respectively. The volume of sodium hydroxide used throughout the hydrolysis was noted for 5 h. The enzymatic activity was terminated by adjusting the pH to 4.0, allowed to cool at 25 °C, and centrifuged at 5000× *g* for 10 min at 4 °C. The supernatant of each fraction (hydrolyzed by each enzyme) was collected and pH-adjusted to 7.0 before being lyophilized.

### 2.7. Degree of Hydrolysis (DH) of Cricket Protein Fractions

The DH (%) of the protein was determined according to the method used in Adler-Nissen [26] using the amount of alkaline (NaOH) consumed. The degree of hydrolysis can be calculated from the following equation:

DH (%) = B × N_b_ × 1/α × 1/MP × 1/h_tot_ × 100 (1)

B represents the volume of NaOH consumed (mL);
N_b_ represents the normality of the NaOH;
MP represents the weight of protein (g) (N × 6.54);
h_tot_ represents the total number of peptide bonds in the protein substrate (8.64 meq/g) [27];
α represents the average degree of dissociation of the α-NH_2_ groups.

α = [(10 pH − pKa)/(1 + 10 pH − pKa)](2)

pKa is the average value of pK (α-amino groups) liberated during hydrolysis.

### 2.8. Antioxidant Activity Determinations

#### 2.8.1. ABTS Radical-Scavenging Activity of Cricket Protein Hydrolysates

With a little adjustment, the ABTS radical-scavenging activity of the protein hydrolysates was determined according to Phongthai et al. [14,22]. The stock solution containing 7.4 mM ABTS and 2.6 mM potassium persulfate (K_2_S_2_O_8_) (1:1) was incubated in the dark for 12 h. The solution was diluted with distilled water in a ratio of 1:60. A volume of 150 µL from the sample solutions was pipetted into a test tube and 2850 µL of the working solution was added to it and vortexed. The mixture was incubated for 2 h in the dark, and absorbance was deduced at a wavelength of 734 nm. A standard curve of mean absorbance (*y*-axis) against the Trolox concentration (0–320 µM) (*x*-axis) was used as the reference (µmol Trolox equivalent/g sample).

#### 2.8.2. Metal-Chelating Activity of Cricket Protein Hydrolysate

The metal-chelating activity of protein hydrolysate was investigated according to the method of Phongthai et al. [22] with some modifications. A volume of 3 mL from the samples was first mixed with 200 µL of 325 µM FeSO_4_. Afterwards, 200 µL of 800 µM ferrozine was added and put in the dark for 10 min. The absorbance was measured at a wavelength of 562 nm. A standard curve was generated by plotting the mean absorbance (*y*-axis) against the ethylenediaminetetraacetic acid (EDTA) concentration (100–250 µM) (*x*-axis). The values were expressed in terms of µmol Trolox equivalent/g sample.

### 2.9. Fractionation and Purification of Antioxidant Peptides

#### 2.9.1. Fractionation of Cricket Protein Hydrolysate by Ultrafiltration

The three CPHs of glutelin (hydrolyzed with Alcalase, Flavourzyme, and Protamex) were further purified because of their high antioxidant activities. The hydrolysates were fractionated following the method of Upata et al. [27]. The hydrolysates were dissolved in DI water (1% *w*/*v*), centrifuged, and filtered with a membrane ultrafiltration system, which comprised Amicon^®^ stirred cells (MERCK, Darmstadt, Germany) using 3 kDa and 10 kDa molecular weight cutoff (MWCO) membrane (to obtain <3 kDa, 3–10 kDa, and >10 kDa microcon) at a flow rate of 2 mL/min for 3 kDa and 10 mL/min for 10 kDa, respectively. The permeate was collected and lyophilized. The permeate from the glutelin hydrolyzed with Alcalase had the highest ABTS activity while that from the glutelin hydrolyzed with Protamex had the highest metal-chelating activity. Both fractions were further purified by using size-exclusion chromatography.

#### 2.9.2. Gel-Filtration Chromatography

The fraction G/A (<3 kDa) from 2.9.1 was separated into distinct fractions using size-exclusion chromatography. A quantity of 100 mg from the sample was dissolved in 2 mL of filtered water and the solution was loaded on a Sephadex G-25 gel filtration column (1.5 × 75 cm) (Versatile Preparative HPLC System LC-Forte/R-II | YMC CO., LTD, Kyoto, Japan) with a flow rate of 5 mL/min. The fraction was eluted by suction mode through the injection of up to 3 mL containing 2 mL of the sample solution and 1 mL of filtered water. Each fraction was collected by using a fraction collector attached to the Versatile Preparative HPLC (LC-forte/R-II; YMC). The absorbances were monitored at 220 nm and 280 nm. Two fractions, GA-1 (<3 kDa) and GA-2 (<3 kDa), were collected and lyophilized. The ABTS and metal-chelating activities of both fractions were determined and the amino acid sequence of the fraction with the highest activity was identified using LC-MS/MS.

#### 2.9.3. Identification of Peptide Sequence by LC-MS/MS

The amino acid sequence of the peptides was identified by LC-MS/MS as described by Narvaez-Rivas et al. [28] with modification. Lyophilized peptide powder from protein hydrolysate was solubilized in 0.1% formic acid in water (0.2 μg/mL). The characteristics of peptidomics were analyzed using Orbitrap HF mass spectroscopy with an ESI ion source operated at 3.2 kV. One microgram of peptide content (5 μL) was loaded on a C18 column (2-technical replication; *n* = 2). The column temperature was maintained at 60 °C during the separation. The mobile phase was composed of water with 0.1% formic acid (A) and acetonitrile with 0.1% formic acid (B). The gradient elution program was as follows: 0 min, 95%A; 15 min, 60%A; 20 min, 20%A; 25 min, 5%A; and 35 min, 95%A. A formic acid/water solution with a concentration of 0.1% was administered after every injection to avoid sample overload between runs. MS spectral data were acquired using a Top 10 method, dynamically choosing the most abundant precursor ions from the wide range of survey scans (150–2000 *m/z*) with charge states (+1 to +5). The dynamic exclusion duration was 20 s. The isolation of precursors was performed with a mass of 1.4 *m/z* and MS/MS scans were acquired with a starting mass of 120 *m/z*. Survey scans were acquired at a resolution of 120 k at *m/z* 400. The resolution for fragmentation spectra was set to 30,000 at *m/z* 200. Normalized collision energy by HCD was set at 29. QCs of the sample preparations and LC-MS/MS parameters were conducted for confirming the reproducibility data. Both chromatographic analysis and MS-acquisition analysis of 2 LC-MS runs were conducted.

#### 2.9.4. De Novo Peptide Sequencing

To discover the amino acid sequence of a peptide given an MS/MS spectrum and the peptide mass, 2 replicate analytical LC-MS runs were achieved by de novo sequencing algorithms using the Peak StudioX software version 10.5 (Bioinformatics Solutions Inc., Waterloo, ON, Canada) [29,30]. The peptide mass tolerance was set to 20 ppm and 0.2 Da for MS/MS. To obtain high-confidence peptide identification, a false discovery rate of 1% was used for identified-peptide filtering. The best 10 ranked-hypotheses peptide sequences with the highest abundances were reported.

### 2.10. Statistical Analysis

The results were reported as means ± standard deviation of triplicate measurements. The analysis of the variance of the results was determined statistically at a 5% significance level using the IBM SPSS software (Version 20).

## 3. Results and Discussion

### 3.1. Osborne Fractionation of Cricket Protein

**Figure 2:**
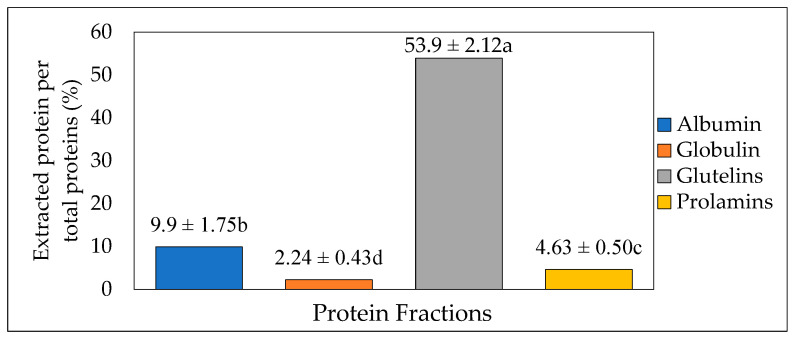
Protein extraction yield (%) from cricket protein using Osborne fractionation method (mean value of 4 batches, each with 200 g of fresh cricket blended with 1000 mL of distilled water). The letters a-d represent the significant differences in the protein fractions *p* < 0.05.

Osborne fractionation was used to extract different fractions of protein from the crickets. The method involves the release of protein fractions based on their solubilities in different solvents (Water, 5% NaCl, 0.1 M NaOH, and 70% ethanol). The total extraction yield recovered was up to 70.6%, whereas glutelin after three steps had the highest protein proportion (53.9%, *p* < 0.05). This was followed by water-soluble albumin, ethanol-soluble prolamin, and NaCl-soluble globulin, with 9.9%, 4.63%, and 2.24% protein proportions, respectively (Figure 2). This result was similar to the result obtained by Caligiani et al. [31] in their research on the extraction of proteins in black soldier fly prepupae. In their stepwise Osborne fractionation, glutelin was the highest fraction obtained, with 31 ± 5%, followed by albumin and globulin, with 23 ± 2% and 14 ± 2%, respectively, while prolamin had the lowest yield, with 9 ± 1%. However, in contrast to the result obtained in the present study, Sardari et al. [17] also observed prolamin as the smallest yield, with 5.1%, compared to glutelin, with 27.9%, from the Osborne fractionation of fine rice bran proteins. The different proportions of these protein fractions are varied depending on the sources of protein. Thus, the maximum extraction yield can be obtained when knowing both a major protein fraction in raw materials as well as the suitable solvent.

Osborne fractionation, employed in the present study, allows for the extraction of protein fractions under mild conditions, which accounts for integrity and the assurance of more intact protein fractions. The results of the present study have shown that the total protein content in cricket protein is not entirely extracted after prolamin. This is because portions of proteins such as membrane proteins and lipoproteins cannot be extracted through Osborne fractionation. After all, they are insoluble and remain in the residue together with starch [32].

### 3.2. Amino Acid Compositions of CPF

The composition of amino acids in a protein sample determines, to an extent, the bioactivity of the present peptides. Generally, hydrophobic amino acids and aromatic amino acids (valine, methionine, isoleucine, leucine, methionine, alanine, tyrosine, tryptophan, and phenylalanine) are present in the most active antioxidant peptides [33]. Table 1 shows the amino acid compositions of CPF in albumin, globulin, glutelin, and prolamin (mg/g). The results in the table show that most of the hydrophobic and aromatic amino acids are abundant in all the fractions, especially in glutelin and prolamin; however, tryptophan is a limiting amino acid in the cricket sample, which agrees with the findings of Köhler et al. [34]. Among the electrically charged amino acids, aspartate and glutamate are also abundant in glutelin and prolamin. The individual amino acid values are comparable to those of the normal animal-based protein sources required for a healthy diet, and the essential amino acids like histidine, threonine, valine, lysine, isoleucine, leucine, phenylalanine, tryptophan, and cysteine in the fractions meet the requirements for human adults as indicated in FAO/WHO reference protein [35]. For example, the amino acid profile of glutelin met the requirement of 40% EAAs. The abundance of those important amino acids is evidence that Cricket Protein Fractions contain antioxidant peptides.

In a study conducted on the amino acid profiles of some selected edible insect species, species-specific deficiencies, notably in leucine, were reported. Nsevolo Miankeba et al. [36] reported that the content of essential and non-essential amino acids varies between insect species; for example, in his study, the contents of Ile, Phe, Thr, and Trp in I. *ertli* (Saturniidae) were highest compared to those in other species.

### 3.3. Protein Pattern of Cricket Protein Fractions Characterized by SDS-PAGE

**Figure 3:**
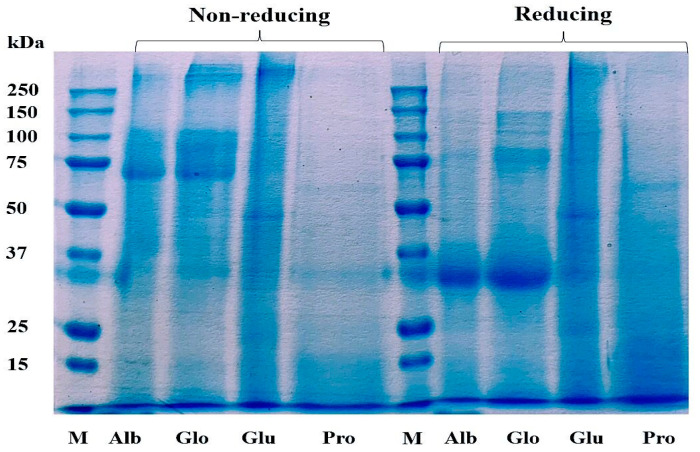
Profile of proteins in Cricket Protein Fractions by SDS-PAGE. M represents the protein marker which is the standard of known molecular weight protein. Alb, Glo, Glu, and Pro represent the four protein fractions albumin, globulin, glutelin, and prolamin, respectively.

The Cricket Protein Fractions were characterized by using the electrophoresis technique as described by Laemmli et al. [25]. Figure 3 shows the protein pattern of the protein fractions. SS bonds play an important role in the structure of storage proteins, and they are formed between the thiol group of the amino acid cysteine in a disulfide linkage that stabilizes the structure of the protein [37]. The chemical modification of proteins can result in the degradation or aggregation of the secondary and tertiary structures through interaction with disulfide bonds and hydrophobic interaction [38].

The protein pattern of albumin, globulin, and glutelin was observed between the range of 50–250 kDa for the non-reducing condition and between 30–250 kDa for the reducing condition. The highest staining was observed in the band that corresponds with a molecular weight of 30 kDa in albumin and globulin, indicating high concentration as a result of the addition of a reducing agent. The protein in glutelin was dominant at the intermediate (50 kDa) and high molecular weights (>250 kDa), with little protein below 30 kDa, which was in agreement with the findings of De Brier et al. [39]. There was no change in the protein pattern of prolamin in both the reducing and non-reducing conditions. This might have been a result of the absence of cysteine in the prolamin fraction (Table 1), which can mean that there is an absence of disulfide bonds in its structure. This result corresponds to the study of Hall et al. [40] and Santiago et al. [41], who described the protein profile of *G. sigillatus* and *G. assimilis* as containing bands of 14.4 kDa to 212 kDa and 10 kDa to 250 kDa, respectively. However, contrary to this study, the protein profile of the cricket proteins was found to be concentrated at 45 kDa.

### 3.4. Estimation of the Degree of Hydrolysis (DH) of CPF

The functional properties and bioactivity of proteins can be modified by adjusting their size and controlling the enzymatic reaction during hydrolysis [42]. The hydrolysis of protein exposes the free amino acids and peptides that are initially in their inactive forms. Physicochemical conditions such as pH, temperature, hydrolysis time, and enzyme inactivation are parameters that are critical to the type of bioactive peptides produced. Hence, the parameters must be controlled and optimized. In most cases during hydrolysis with enzymes, albumin and globulin are susceptible to hydrolysis whereas glutelin and prolamin are resistant. The susceptibility of albumin and globulin might be associated with the formation of complexes with other compounds, which are easily released during digestion [43]. Moreover, the antioxidant activity of most hydrolysates increases with hydrolysis [44]. However, it varies with different protein sources.

**Figure 4:**
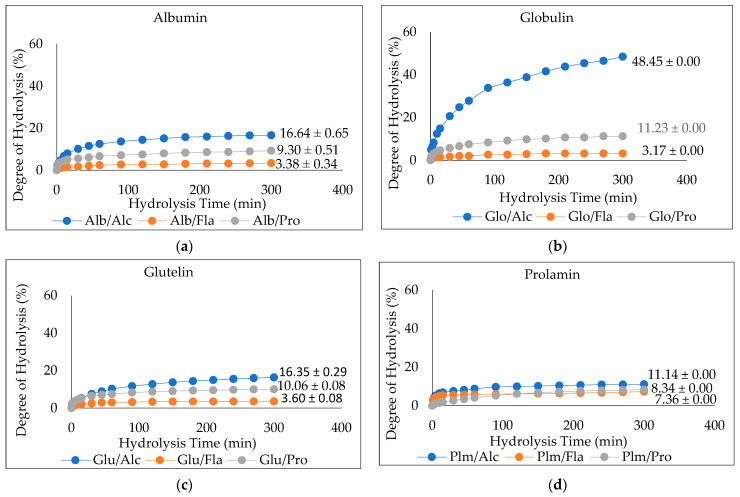
(**a**–**d**). Degree of Hydrolysis (DH) of CPF by Proteases (Alcalase, Flavourzyme, and Protamex): Alcalase at pH 8.5, 55 °C, Flavourzyme at pH 7.0, 50 °C, and Protamex at pH 7.0, 50 °C for 5 h.

**Figure 5:**
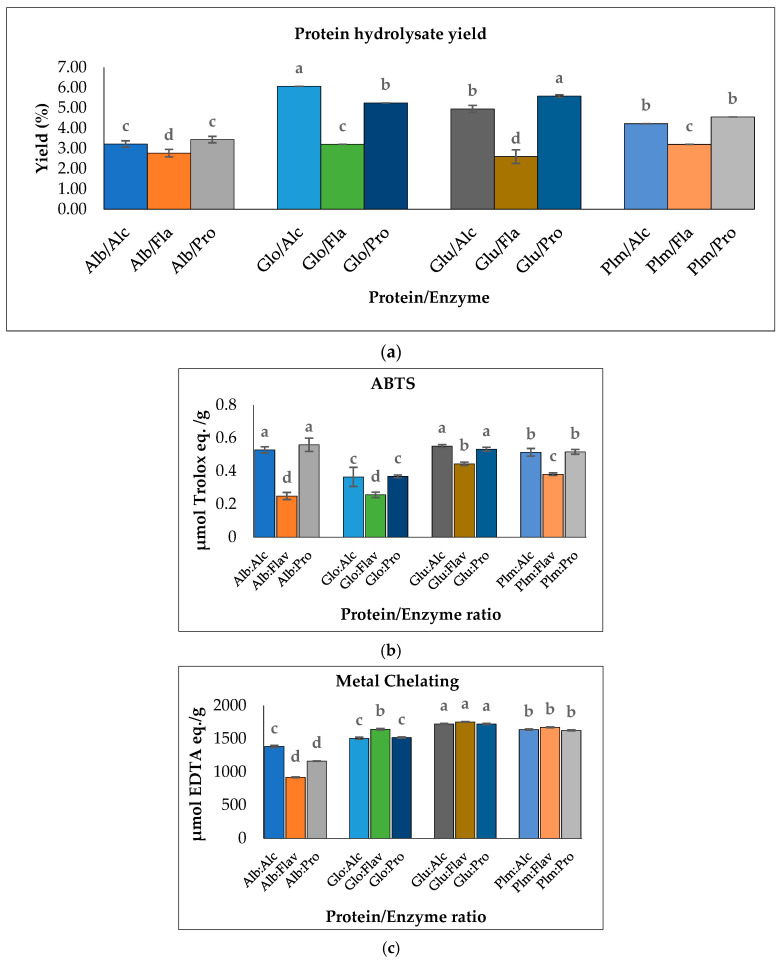
(**a**) Hydrolysate yield of Cricket Protein Fractions; (**b**) ABTS radical scavenging activities; (**c**) metal-chelating activities of Cricket Protein Fractions at a concentration of 1 mg/mL. All data were reported as mean ± SD (*n* = 2) and indicated statistical difference (*p* < 0.05). The letters a–d represent the significant differences in the Protein/Enzyme fractions *p* < 0.05.

The degree of hydrolysis (DH) of the enzymatically hydrolyzed Cricket Protein Fractions was investigated. Figure 4a–d shows the DH of albumin, globulin, glutelin, and prolamin enzymatically hydrolyzed with proteases (Alcalase, Flavourzyme, and Protamex), while Figure 5a shows their respective hydrolysate yield. The results show that the hydrolysates prepared with Alcalase had the highest degree of hydrolysis, with the highest observed in globulin at 48.45 ± 0.00% (*p* < 0.05) compared to the other enzymes in each protein fraction. In general, the present Alcalase, Flavourzymes, and Protamex were able to cleave peptide bonds in the Cricket Protein Fractions, but to varying extents. Alcalase has the highest degree of hydrolysis across all fractions, followed by Protamex and Flavourzyme, which implies the effectiveness of alkaline proteases over neutral proteases. The result was consistent with the results of Cui et al. [45]. Alcalase is an alkaline serine endopeptidase derived from bacteria and can easily hydrolyze protein. The enzyme has been observed to cleave peptide bonds on the carboxyl side of Glu, Met, Leu, Tyr, Lys, and Gln [46,47]. All this specificity accounts for its high degree of hydrolysis. On the other hand, Flavourzyme is an endopeptidase, and partially an exopeptidase, that is mainly involved in the release of free amino acids. It has the potential of producing amino acids with ionizable C-terminal carboxylic and N-terminal amino groups with low concentrations [48]. Protamex is an endopeptidase like Alcalase [49], but its efficacy to hydrolyze a cricket protein in the study was lower than that of Alcalase. The difference in the degree of hydrolysis can also be better and further explained by the various hydrolytic sites, enzyme specificity and affinity, and substrate composition [50].

The results also showed that the proteins hydrolyzed by Flavourzyme had the lowest degree of hydrolysis and the lowest ABTS radical-scavenging activity (Figure 5b). In contrast, Flavourzyme had the highest metal-chelating activity in globulin, glutelin, and prolamin (Figure 5c). Globulin hydrolyzed with Alcalase had the highest degree of hydrolysis: up to 48.45% (*p* < 0.05). However, to ensure peptide uniformity and reproductivity, it is advised that a hydrolysate with a moderate degree of hydrolysis should be used for the preparation of functional peptides [44]. Glutelin had a moderate degree of hydrolysis within the range of 3–15% DH and the antioxidant activities of its hydrolysates were the highest (Figure 5b,c). Moreover, the yield was substantial—almost in the same range as that of globulin. Glutelin has the highest ABTS radical-scavenging activity and metal chelating antioxidant activity, which can be as a result of its high hydrophobic amino acid content, as amino acids have a major role to play in the bioactivity of hydrolysates. This, together with other factors such as the hydrolysate yield and degree of hydrolysis, led to the selection of glutelin for further protein purification.

### 3.5. Purification of Glutelin Hydrolysates

**Figure 6:**
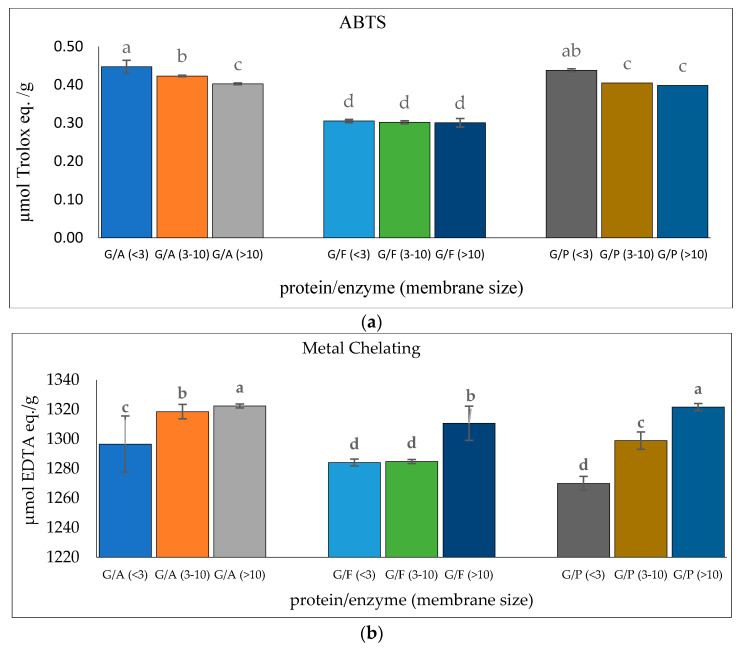
(**a**) ABTS radical-scavenging activity of cricket protein hydrolysate after membrane ultrafiltration; (**b**) metal-chelating activity of cricket protein hydrolysate after membrane ultrafiltration at a concentration of 1 mg/mL. All data were reported as mean ± SD (*n* = 2) and indicated statistical difference (*p* < 0.05). The letters a–d represent the significant differences in the protein fractions *p* < 0.05.

Glutelin was further purified through a series of purification stages. In this context, glutelin was passed through membrane ultrafiltration to obtain <3 kDa, 3–10 kDa, and >10 kDa microcon. The ABTS radical-scavenging activity and metal-chelating activity of the glutelin fractions were determined for the most active fraction. Glutelin samples hydrolyzed with Alcalase (GA <3 kDa) had the highest ABTS activity with 0.45 µmol Trolox eq./g while GA (>10 kDa) had the highest metal-chelating activity with 1322.38 µmol EDTA eq./g (*p* < 0.05), as shown in Figure 6. There is speculation that the high antioxidant activity of GA (<3 kDa) and GA (>10 kDa) is a result of the binding ability of the ultra-filtered fractions, their high amino acid composition, and their containing high amounts of hydrophobic amino acids [51]. Previous research has shown that the lowest peptide fractions with the lowest molecular weights are often the ones with the highest bioactivity. This can be attributed to the presence of simple peptide sequences that allows the binding of functional groups to target protein or free radicals [52]. GA (<3 kDa) and GA (>10 kDa) were selected for further purification using size exclusion chromatography because they both had the highest ABTS antioxidant activities and metal-chelating activities.

### 3.6. Antioxidant Activity of Peptide Fraction from Gel-Filtration Chromatography (SEC)

To improve the bioactivity of the cricket protein hydrolysate and subsequent identification of the amino acid sequence, the glutelin hydrolysate with the highest ABTS, G/A (<3 kDa), was further purified using size exclusion or gel filtration chromatography. Gel filtration chromatography is distinct for its simplicity and ability to fractionate proteins (peptides) to obtain the most active fraction [16]. It also has the advantage of preventing sample loss and allowing an accurate separation [53]. However, Cermeño et al. [54] have raised concerns about limited peak capacity, low resolution, and consumption of large volumes of the eluent.

**Figure 7:**
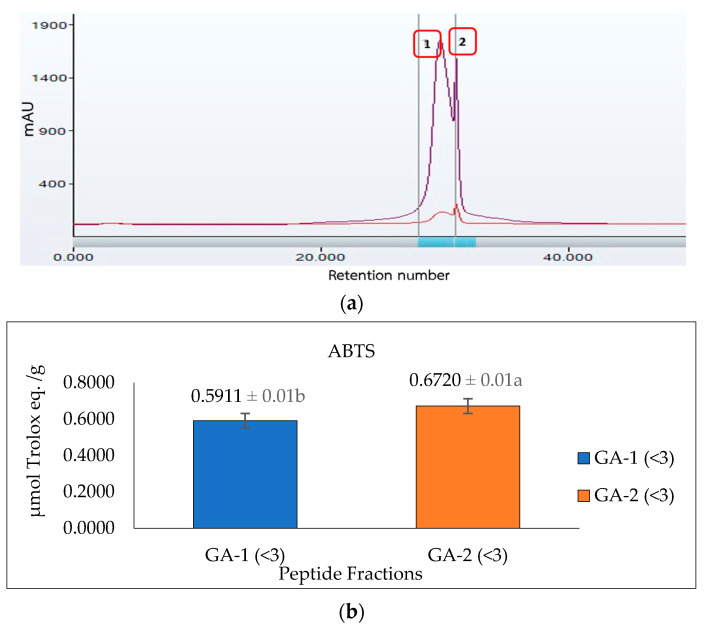

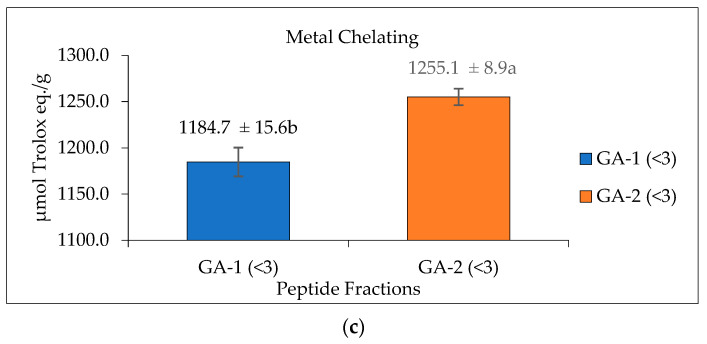
Glutelin purification by size exclusion chromatography. (**a**) Separation of glutelin hydrolysates by using gel filtration chromatography on a Sephadex G-25 column into two fractions (1 and 2) at a flow rate of 5 mL/min. The eluted fractions were detected at 220 nm and 280 nm; Fraction 1 represents GA-1 (<3 kDa) while fraction 2 represents GA-2 (<3 kDa) (**b**) ABTS radical-scavenging activity of the eluted fraction from the column with a concentration of 50 mg/mL; (**c**) metal-chelating activity of the eluted fraction from the column with a concentration of 50 mg/mL. All data were reported as mean ± SD (*n* = 2) at *p* < 0.05. The letters a and b represent the significant differences in the protein fractions *p* < 0.05.

Glutelin hydrolysate hydrolyzed by Alcalase with <3 kDa from Section 3.5 was loaded to a Sephadex G-25 column (Figure 7a) to separate it into two fractions (1 and 2) at a flow rate of 5 mL/min. Two peaks were separated and collected namely GA-1 (<3 kDa) and GA-2 (<3 kDa). The two fractions showed ABTS radical-scavenging activity of 0.5911 and 0.6720 µmol Trolox eq./g and metal-chelating activity of 1184.7 and 1255.1 µmol Trolox eq./g, respectively. GA-2 (<3 kDa), with the lowest retention time and lowest molecular weight, showed the highest ABTS radical-scavenging activity (0.6720 µmol Trolox eq./g) (Figure 7b) and metal-chelating activity (1255.1 µmol Trolox eq./g) (Figure 7c) at a concentration of 50 mg/mL. This result corresponds with previous results that reported that peptides from perilla (*Perilla frutescens,* L. Britton) seed protein hydrolysates with smaller molecular weights have higher antioxidant activities compared with those with higher molecular weights [55]. However, from the result obtained from the antioxidant activities of glutelin in both the ABTS and metal-chelating assays, it can be concluded that there is a synergistic effect on the hydrolysate fraction that is responsible for the high activity of the hydrolysate fraction compared to the fractionated samples. Aside from the size and molecular weight of the peptide, the type of amino acid and the peptide sequence are also key determinants of the ability of the peptide to scavenge free radicals, particularly hydrophobic amino acids that have been linked to higher antioxidant activity [55,56].

### 3.7. Identification of Antioxidant Peptide Sequence by LC-MS/MS

**Figure 8:**
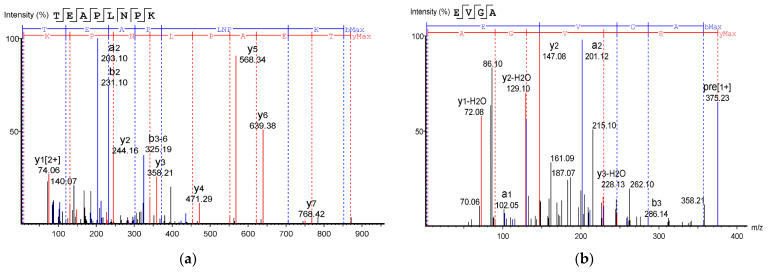

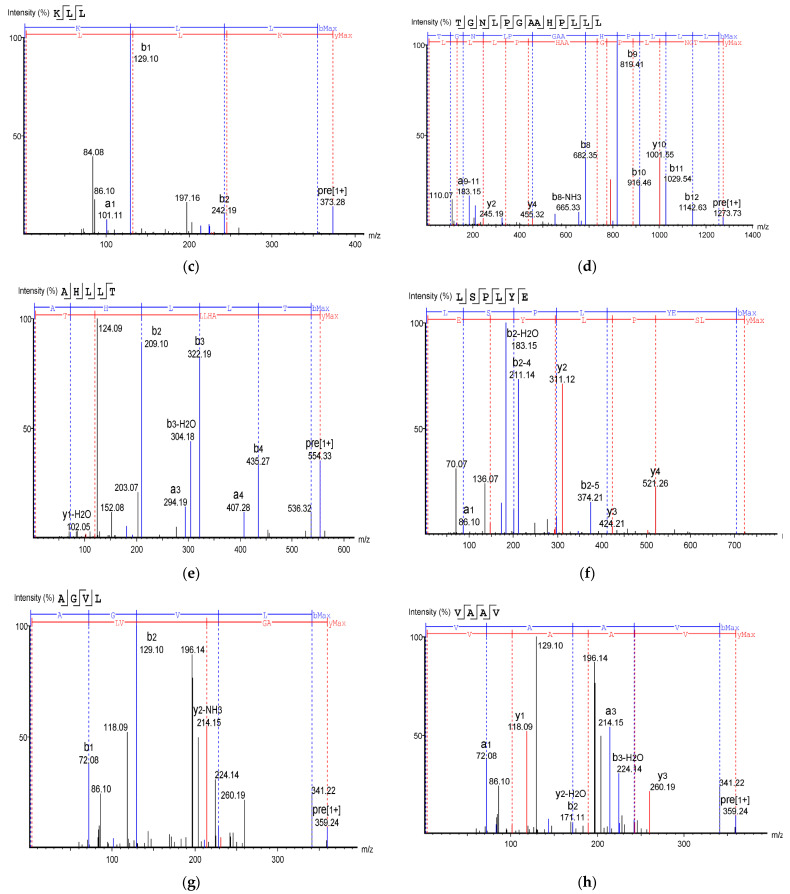

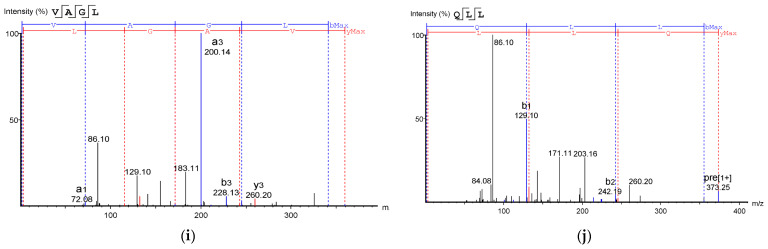
Mass Spectrum of 10 selected antioxidant peptides from cricket protein (*Gryllus bimaculatus*): (**a**) TEAPLNPK; (**b**) EVGA; (**c**) KLL; (**d**) TGNLPGAAHPLLL; (**e**) AHLLT; (**f**) LSPLYE; (**g**) AGVL; (**h**) VAAV; (**i**) VAGL; (**j**) QLL. [(**a**–**e**): GA-1 (<3); (**f**–**j**): GA-2 (<3)].

Table 2 shows the identified antioxidant peptides from the glutelin hydrolysate fraction of cricket protein. The sequences found in GA-1 (<3 kDa) were TEAPLNPK, EVGA, KLL, TGNLPGAAHPLLL, and AHLLT, while those found in GA-2 (<3 kDa) were LSPLYE, AGVL, VAAV, VAGL, and QLL. Figure 8 represents the mass spectra of 10 selected antioxidant peptides, and the molecular weights of the peptides were within the range of 359.23–721.37 Da. Moreover, the mass spectra of the ions aligned with the ions in the mascot database, indicating the accuracy of the database. All the peptides identified contained hydrophobic and essential amino acids including alanine, leucine, and valine. Overall, the hydrophobic amino acids in the identified peptide sequences ranged from 50–100% of the whole amino acid in the sequence. It has been reported that peptide sequences with hydrophobic amino acids and aromatic amino acids exhibit strong antioxidant potential [57], and that a larger number of amino acids such as Gly, Pro, and Leu accounts for the total amino acid content in bioactive peptides [56]. Hydrophobic amino acids can enhance peptide solubility and scavenge free radicals [58]. The peptides from GA-2 (<3 kDa) contain tyrosine, valine, and leucine, which explains its high antioxidant activity. Many bioactive peptides are closely related to their amino acid composition, which allows them to donate hydrogen or electron to undergo radical scavenging activities [59]. Sae-Leaw et al. [60] observed that a high level of hydrophobic amino acids with repeating Gly-Pro sequences in peptides derived from gelatin hydrolysate of seabass skin possessed high ABTS radical-scavenging activity. In this study, they identified peptide chains with 5–12 amino acids containing hydrophobic amino acids in concentration of between 50–100%, which correlates with the findings of the present study. Amino acid sequences containing hydrophobic amino acids like alanine, valine, and leucine have strong antioxidant activity against attacks and contribute to improving peptides’ bioactivity [59,61]. Najafian and Babji [62] revealed, in their identification of bioactive peptides from *Pangasius sutchi,* that proline, valine, histidine, and aspartic acid are present in the peptides identified. Their study also showed that valine and leucine are types of hydrophobic amino acids that can enhance peptides in water–lipid interaction to facilitate free radical-scavenging activity. The prevalence of hydrophobic amino acids in the amino acid sequence of a peptide increases their antioxidant activity [44]. Intarasirisawat et al. [19] emphasized the effect of hydrophobic amino acids in antioxidant peptides, noting that the hydrophobic amino acids making up between 50–100% of the identified peptides from skipjack (*Katsuwana pelamis*) roe exhibited high ABTS radical-scavenging activity. However, they also found that the peptide sequence with the highest hydrophobic amino acid portion (up to 80% (MLVFAV)) exhibited low ABTS radical-scavenging activity. This was probably due to the improper configuration of the peptide to scavenge ABTS radicals.

Generally, the active peptides containing hydrophilic amino acids such as histidine, serine, and glutamate play a key role in chelating metal ions because of their ability to entrap transition metals [63,64]. However, in this study, the peptides fraction with higher hydrophobicity showed higher metal-chelating activity. This may be attributed to other related factors such as the position of amino acids in the sequences or even the molecular weights of the peptides. For instance, GA-2, which had a smaller molecular weight (avg. 434.46 Da), showed significantly higher metal-chelating activity than GA-1 (avg. 475.08 Da) (*p* < 0.05). This result is consistent with the study of Li et al. [65] who stated that peptides from corn gluten meal hydrolysates with molecular weight between 500–1500 Da have stronger antioxidant activity than peptides with molecular weight above 1500 Da. Overall, amino acids occurring in the sequence of peptides, peptide configuration, and molecular weight may individually influence and/or synergize to promote their antioxidant activities. However, the bioactive functionality of a peptide will depend on how much of it will resist the digestive process and reach the target tissue. Therefore, the functionality tested in vitro will not always reflect the same result in vivo.

## 4. Conclusions

Crickets have high potential to be used as an alternative source of protein and bioactive peptides. The extraction yield of cricket protein was up to 70.6%, and glutelin had the highest protein proportion, followed by albumin, prolamin, and globulin. All derived protein fractions seemed to compose of suitable substrates for Alcalase, particularly the globulin fraction, as the highest degree of hydrolysis was obtained. The antioxidant screening of the hydrolysates shows that glutelin hydrolysate by Alcalase has high antioxidant activity in addition to its high protein content, which makes it selected for further separation with gel filtration chromatography to separate the hydrolysate into fractions. The amino acid sequence of the peptides shows the prevalence of hydrophobic amino acids such as valine and leucine, including tyrosine in the peptide chains, which accounts for its high antioxidant activity. Our study reveals that glutelin fraction in cricket protein is a good source of edible bioactive peptides that can be further applied in functional food products such as beverages and nutraceutical products.

## Figures and Tables

**Table 1:** Amino acid composition of Cricket Protein Fractions (CPF).

Amino Acids		Protein (mg/g)			
		Albumin	Globulin	Glutelin	Prolamin
Hydrophilic	Aspartate (Asp)	16.30	20.60	54.70	63.50
AA	Glutamate (Glu)	22.40	26.70	63.40	75.90
	Arginine (Arg)	9.80	12.50	30.00	33.80
	Histidine (His)	3.20	4.00	10.10	10.50
	Threonine (Thr)	6.80	8.50	19.30	20.90
	Serine (Ser)	7.70	9.60	27.60	24.90
Amphipathic	Cysteine (Cys)	0.50	0.50	0.20	0
AA	Lysine (Lys)	10.10	13.30	28.10	27.10
	Tyrosine (Tyr)	4.60	6.60	18.40	20.50
	Methionine (Met)	0	0.10	5.30	6.20
Hydrophobic	Alanine (Ala)	10.80	13.40	28.00	31.20
AA	Glycine (Gly)	7.10	7.70	14.70	15.40
	Valine (Val)	8.60	10.80	26.70	28.90
	Proline (Pro)	6.60	7.60	16.00	19.20
	Isoleucine (Ile)	5.90	7.70	19.50	21.50
	Leucine (Leu)	12.80	16.90	39.80	45.10
	Phenylalanine (Phe)	6.10	7.80	19.70	20.40

**Table 2:** Antioxidant peptides derived from crickets (in glutelin fraction), identified by LC-MS/MS.

Sample	Peptides	Amino Acid Sequence	Molecular Weight (Da)	% Hydrophobic AA in Sequence
GA-1	TEAPLNPK	Thr-Glu-Ala-Pro-Leu-Asn-Pro-Lys	435.24	50
(<3 kDa)	EVGA	Glu-Val-Gly-Ala	375.19	75
	KLL	Lys-Leu-Leu	373.28	67
	TGNLPGAAHPLLL	Thr-Gly-Asn-Leu-Pro-Ala-Ala-His-Pro-Leu-Leu-Leu	637.37	75
	AHLLT	Ala-His-Leu-Leu-Thr	554.33	60
GA-2	LSPLYE	Leu-Ser-Pro-Leu-Tyr-Glu	721.37	50
(<3 kDa)	AGVL	Ala-Gly-Val-Leu	359.23	100
	VAAV	Val-Ala-Ala-Val	359.23	100
	VAGL	Val-Ala-Gly-Leu	359.23	100
	QLL	Gln-Leu-Leu	373.24	67

## Data Availability

Data are contained within the article.
